# Cellular models and therapeutic perspectives in hypertrophic cardiomyopathy

**DOI:** 10.1515/medgen-2021-2094

**Published:** 2021-12-03

**Authors:** Gökhan Yigit, Bernd Wollnik

**Affiliations:** Institute of Human Genetics, University Medical Center Göttingen, Heinrich-Düker-Weg 12, 37073 Göttingen, Germany; DZHK (German Centre for Cardiovascular Research), partner site Göttingen, Göttingen, Germany; Cluster of Excellence “Multiscale Bioimaging: From Molecular Machines to Networks of Excitable Cells” (MBExC), University of Göttingen, Göttingen, Germany

**Keywords:** hypertrophic cardiomyopathy, induced pluripotent stem cells, engineered heart tissue, genome editing, CRISPR/Cas9

## Abstract

Hypertrophic cardiomyopathy (HCM) is a clinically heterogeneous cardiac disease that is mainly characterized by left ventricular hypertrophy in the absence of any additional cardiac or systemic disease. HCM is genetically heterogeneous, inherited mainly in an autosomal dominant pattern, and so far pathogenic variants have been identified in more than 20 genes, mostly encoding proteins of the cardiac sarcomere. Based on its variable penetrance and expressivity, pathogenicity of newly identified variants often remains unsolved, underlining the importance of cellular and tissue-based models that help to uncover causative genetic alterations and, additionally, provide appropriate systems for the analysis of disease hallmarks as well as for the design and application of new therapeutic strategies like drug screenings and genome/base editing approaches. Here, we review the current state of cellular and tissue-engineered models and provide future perspectives for personalized therapeutic strategies of HCM.

## Introduction

Hypertrophic cardiomyopathy (HCM) is the most frequently occurring inherited disorder of the heart. Worldwide, the prevalence of HCM is estimated between 1:500 and 1:1000 in the general population, independent of sex and ethnic origin [1]. The clinical diagnosis of HCM is based on morphological detection of left ventricular (LV) wall hypertrophy without dilatation of the ventricle and/or other cardiac or systemic diseases [2]. In adults, a wall thickness of ≥15 mm in one or more LV myocardial segments indicates HCM, but the extent of thickening of the LV myocardium and its exact location can be highly variable, and familial cases with lesser degrees of LV wall thickening (13–14 mm) have been described. Additional clinical findings may include mild right ventricular hypertrophy, morphologic anomalies of the mitral valve, myocardial fibrosis, and electrocardiographic abnormalities [3], [4]. HCM is considered an important cause of atrial fibrillation and heart failure in young and previously asymptomatic patients, making it the major cause of sudden cardiac death in the young population [5].

HCM is a genetically heterogeneous disorder with several hundreds of pathogenic variants in more than 28 genes/loci identified to date (Table 1) [6], [7]. These genes mainly encode components of the cardiac sarcomere or the adjacent Z-disc. Most of the known genetic causes of HCM show an autosomal dominant pattern of inheritance, although single cases with autosomal recessive inheritance have also been described to be clinically associated with an earlier disease onset [8], [9]. Currently, in 60–70 % of adolescents and adults with HCM pathogenic variants in cardiac sarcomere genes can be identified [7]. Among these, pathogenic variants in *MYH7*, encoding the β-myosin heavy chain, and *MYBPC3*, encoding the cardiac myosin-binding protein C, are the most frequently found, accounting in total for about 70 % of patients in whom pathogenic variants can be identified. In the remaining patients, variants in genes encoding additional sarcomere components (i. e., *ACTC1*, *ACTN2*, *MYL2*, *MYL3*, *MYOZ2*, *TNNI3*, *TNNT2*, and *TPM1*) can be detected, with each gene accounting for 1–5 % of cases [7]. Additionally, HCM may also occur as part of a metabolic disorder, like Fabry disease, or a congenital syndrome, as observed, e. g., in patients with RASopathies (such as Noonan, Noonan-related, and cardio-facio-cutaneous syndromes), which can escape detection in early childhood due to the mild phenotypic presentation of the underlying syndrome at the time when HCM is diagnosed [10].

**Table 1:** List of genes associated with hypertrophic cardiomyopathy.

CMH	OMIM (phenotype)	Gene	OMIM (gene)	Chromosome	Protein	Inheritance
CMH	192600	*CAV3*	601253	3p25.3	Caveolin 3	AD, DD
CMH1	192600	*MYH7*	160760	14q11.2	Myosin heavy chain 7	AD, DD
CMH1 (DD)	192600	*MYLK2*	606566	20q11.21	Myosin light chain kinase 2	AD, DD
CMH2	115195	*TNNT2*	191045	1q32.1	Troponin T2, cardiac type	AD
CMH3	115196	*TPM1*	191010	15q22.2	Tropomyosin 1	AD
CMH4	115197	*MYBPC3*	600958	11p11.2	Myosin binding protein C3	AD, AR
CMH6	600858	*PRKAG2*	602743	7q36.1	Protein kinase AMP-activated non-catalytic subunit gamma 2	AD
CMH7	613690	*TNNI3*	191044	19q13.42	Troponin I3, cardiac type	AD
CMH8	608751	*MYL3*	160790	3p21.31	Myosin light chain 3	AD, AR
CMH9	613765	*TTN*	188840	2q31.2	Titin	AD
CMH10	608758	*MYL2*	160781	12q24.11	Myosin light chain 2	AD
CMH11	612098	*ACTC1*	102540	15q14	Actin Alpha Cardiac Muscle 1	AD
CMH12	612124	*CSRP3*	600824	11p15.1	Cysteine and glycine rich protein 3	AD
CMH13	613243	*TNNC1*	191040	3p21.1	Troponin C1, slow skeletal and cardiac type	AD
CMH14	613251	*MYH6*	160710	14q11.2	Myosin heavy chain 6	AD
CMH15	613255	*VCL*	193065	10q22.2	Vinculin	AD
CMH16	613838	*MYOZ2*	605602	4q26	Myozenin 2	AD
CMH17	613873	*JPH2*	605267	20q13.12	Junctophilin 2	AD
CMH18	613874	*PLN*	172405	6q22.31	Phospholamban	AD
CMH20	613876	*NEXN*	613121	1p31.1	Nexilin F-actin binding protein	AD
CMH21	614676	?	614676	7p12.1-q21	–	AD
CMH22	615248	*MYPN*	608517	10q21.3	Myopalladin	AD
CMH23	612158	*ACTN2*	102573	1q43	Actinin alpha 2	AD
CMH24	601493	*LDB3*	605906	10q23.2	LIM domain binding 3	AD
CMH25	607487	*TCAP*	604488	17q12	Titin-cap (Telethonin)	AD
CMH26	617047	*FLNC*	102565	7q32.1	Filamin C	AD
CMH27	618052	*ALPK3*	617608	15q25.3	Alpha kinase 3	AR
CMH28	619402	*FHOD3*	609691	18q12.2	Formin homology 2 domain containing 3	AD

AD, autosomal dominant; AR, autosomal recessive; DD, digenic dominant; CMH, cardiomyopathy hypertrophic.

Genetic testing and data interpretation in HCM is challenging and often complicated by variable penetrance and expressivity that are frequently observed. Further, high-throughput genetic testing often identifies novel variants for which pathogenicity and involvement in the disease is unsolved, and data interpretation is further complicated by the observation that different mutations in the same causative gene can result in different patterns of inheritance (Table 1) [8], [9].

Due to the clinical and genetic complexity of HCM, it became apparent very early that disease models were needed which allow analysis of the heart’s intricate physiology and the pathology leading to HCM and in addition provide experimental platforms for pharmacological compound screenings and development of novel treatment options. Initial studies largely relied on cardiac muscle biopsies that were either derived from post-mortem human heart muscles or HCM patients who underwent surgical myectomy to reduce excess muscle from the thickened wall [11]. These tissue preparations were either directly used, e. g., to perform histological analyses of the cardiac tissue architecture, or enzymatically digested or mechanically dissociated to generate isolated human cardiomyocytes. By means of these tissue preparations several hallmarks of HCM pathology were revealed, including interstitial fibrosis, myocyte enlargement, and structural cellular disarrangements [12]. Isolated and dissociated cardiomyocytes provide a complementary model system providing important insights into how electrophysiological, contractile, and morphological properties are changed in HCM [13], [14]. Still, studies based on cardiac muscle biopsies are limited by access to patient samples. The low availability together with the fact that primary cardiomyocytes derived from tissue samples do not proliferate and tend to dedifferentiate in *in vitro* culture raised the need of additional cellular and experimental models to study human heart physiology and the pathology of HCM [15]. Reprogramming somatic cells into human induced pluripotent stem cells (hiPSCs) and subsequent differentiation of these cells into cardiomyocytes provided a novel source of cardiomyocytes and opened the field for new suitable strategies to overcome previous limitations and to study cardiomyocyte function in health and disease.

## hiPSC-based cellular models of HCM

The development of iPSC-based technologies and the establishment of protocols for differentiation of iPSCs to cardiomyocytes generated a virtually unlimited cell source for detailed modeling and analysis of human cardiac diseases. hiPSCs can be derived from somatic tissue of healthy individuals as well as patients, thus providing disease- and mutation-specific models for characterizing cardiac processes *in vitro*. Differentiation of hiPSCs can be carried out using a variety of different protocols which generally recapitulate cardiac development *in vivo* using signaling factors that take part in physiological differentiation [16], [17]. These include the major signaling pathways WNT, TGFβ, and BMP, which have been identified as crucial pathways involved in cardiac differentiation of hiPSCs into the cardiac lineages [16], [17]. Differentiation steps include mesoderm induction, cardiac mesoderm induction, generation of cardiac progenitors, and formation of cardiomyocytes; the first spontaneously contracting hiPSC-derived cardiomyocytes (hiPSC-CMs) can be observed 8 days after induction of differentiation. hiPSC-CMs expressing sarcomeric components and ion channels show action potentials and cardiac calcium transients, enabling characterization of not only cardiomyocyte structure, but also contractile functions, intracellular Ca^2+^ transients, metabolism, and electrophysiological properties. Additionally, optimized protocols allow efficient differentiation of hiPSCs into highly homogeneous populations of subtype-specific cardiomyocytes, like, e. g., atrial and ventricular hiPSC-CMs, thereby providing suitable models to analyze subtype-restricted mechanisms in cardiac diseases and to develop ventricle-specific drugs [18], [19], [20].

During the last years, hiPSC-CMs have been extensively used to improve our understanding of physiological processes in cardiomyocytes and to characterize the pathological effects caused by genetic variants not only in HCM, but also in other heart diseases, like, e. g., dilated cardiomyopathy and long QT syndrome. Cellular phenotypes observed in these hiPSC-CMs recapitulate the morphological, electrophysiological, and contractile features observed in isolated heart muscle preparation of patients quite well [21].

The first hiPSC-derived modeling of HCM was performed in 2013 by Lan et al., who showed that *in vitro* analyses of hiPSC-CMs derived from HCM patients carrying the pathogenic variant p.Arg633His in *MYH7* revealed features similar to those seen in the HCM disease phenotype [22]. Morphologically, Lan et al. observed (i) a higher proportion of multinucleated cells and an increase in cell size in patient-derived hiPSC-CMs compared to controls and (ii) Ca^2+^-induced arrhythmias which could be mitigated by continuous treatment of cells with the Ca^2+^ channel blocker verapamil [22]. Han et al. confirmed the effects of pathogenic *MYH7* variants on the size and multinucleation of hiPSC-CMs observed by Lan et al. and reported an increased number of patient-derived hiPSC-CMs displaying myofibrillar disarray and disrupted sarcomeres with disorganized Z-lines compared to control hiPSC-CMs (47 % vs. 17 %) [23]. They did not observe an increased ratio of delayed afterdepolarizations (DAD), which was reported by Lan et al., but instead revealed a prolonged action potential duration, suggesting variant-specific cellular effects based on the different functional domains affected by the identified variants in *MYH7* [22], [23].

## hiPSC models in RASopathies

Since then, different studies have been performed describing cellular cardiomyocyte phenotypes in hiPSC-CMs from patients with genetically caused HCM (e. g., due to pathogenic variants *MYBPC3* or *TPM1*) as well as monogenic diseases associated with HCM [24], [25], [26], [27]. Among these, several hiPSC-CM models have been established and characterized especially for RASopathy spectrum disorders including Noonan syndrome with multiple lentigines (formerly referred to as LEOPARD syndrome, OMIM #151100), cardio-facio-cutaneous syndrome (CFC1, OMIM #115150), and autosomal dominant and autosomal recessive Noonan syndrome (NS, OMIM #163950 for autosomal dominant NS1; OMIM #605275 for autosomal recessive NS2) (Table 2) [28], [29], [30], [31].

**Table 2:** List of Noonan syndrome-associated genes.

NS	OMIM (phenotype)	Gene	OMIM (gene)	Chromosome	Protein	Inheritance
NS1	163950	*PTPN11*	176876	12q24.13	Protein tyrosine phosphatase non-receptor type 11	AD
NS2, NS10	605275; 616564	*LZTR1*	600574	22q11.21	Leucine zipper like transcription regulator 1	AR (AD for NS10)
NS3	609942	*KRAS*	190070	12p12.1	KRAS protooncogene, GTPase	AD
NS4	610733	*SOS1*	182530	2p22.1	SOS Ras/Rac guanine nucleotide exchange factor 1	AD
NS5	611553	*RAF1*	164760	3p25.2	Raf-1 protooncogene, serine/threonine kinase	AD
NS6	613224	*NRAS*	164790	1p13.2	NRAS protooncogene, GTPase	AD
NS7	613706	*BRAF*	164757	7q34	B-Raf protooncogene, serine/threonine kinase	AD
NS8	615355	*RIT1*	609591	1q22	Ras like without CAAX 1	AD
NS9	616559	*SOS2*	601247	14q21.3	SOS Ras/Rho guanine nucleotide exchange factor 2	AD
NS11	618499	*MRAS*	608435	3q22.3	Muscle RAS oncogene homolog	AD
NS12	618624	*RRAS2*	600098	11p15.2	RAS related 2	AD
NS13	619087	*MAPK1*	176948	22q11.22	Mitogen-activated protein kinase 1	AD
NS-like disorder with loose anagen hair 2	617506	*PPP1CB*	600590	2p23.2	Protein phosphatase 1 catalytic subunit beta	AD
NS-like with loose anagen hair 1	607721	*SHOC2*	602775	10q25.2	SHOC2 leucine rich repeat scaffold protein	AD
NS-like phenotype	–	*SPRED2*	609292	2p14	Sprouty related EVH1 domain containing 2	AR

AD, autosomal dominant; AR, autosomal recessive; NS, Noonan syndrome.

Noonan syndrome with multiple lentigines is an autosomal dominant disorder caused by pathogenic variants in *PTPN11*, *RAF1*, *BRAF*, and *MAP2K1*. Approximately 85 % of affected individuals have heart defects, with more than 70 % of these patients presenting with HCM [32]. hiPSC-CMs derived from two independent patients carrying the heterozygous pathogenic variant p.Thr468Met in PTPN11 showed a significant increase in cell size and a higher proportion of cells with nuclear NFATC4 localization (80 % vs. 30 %), a key regulator of cardiac hypertrophy, compared to hiPSC-CMs derived from a healthy brother of one of the patients, recapitulating the HCM observed in patients with Noonan syndrome with multiple lentigines [28]. Similar results were obtained by analyzing hiPSC-CMs derived from patients with CFC syndrome [30]. Patient-specific hiPSC-CMs carrying two different pathogenic variants in BRAF (p.Gln257Arg and p.Thr599Arg, respectively) were more than three times larger than control cells, had a pro-hypertrophic gene expression pattern, and showed an increased frequency of irregular Ca^2+^ transients (28 % vs. 6 %). Interestingly, these cellular effects were also observed in hiPSC-CMs of a CFC1 patient who did not show any clinical signs of HCM [30]. Generating hiPSC-CMs from a patient with autosomal dominant Noonan syndrome, Jaffre et al. showed that the pathogenic variants in RAF1 lead to a larger cell size and disorganized myofibrillar structures, and they provided evidence that cardiomyocyte hypertrophy might be driven by increased activity of ERK5 [29]. Similarly, in a model for autosomal recessive Noonan syndrome caused by biallelic variants in *LZTR1*, patient-derived iPSC-CMs were larger and showed dysregulation of Ca^2+^ transients, the latter being pharmacologically treatable with the calcium antagonist verapamil [31]. Interestingly, structural disarrangements of the sarcomeres, as seen in other iPSC-CM models of Noonan spectrum disorders and familial HCM, were not observed [31].

## Genome editing in hiPSC models of HCM

The development of genome editing tools, especially the discovery and application of clustered regularly interspaced short palindromic repeats (CRISPR)/CRISPR-associated protein 9 (Cas9)-based technologies, turned out to act highly synergistic with the development of iPSCs. This has dramatically accelerated the generation of novel cellular models that not only extended our understanding of disease mechanisms and novel therapeutic options, but may also be used to develop novel personalized therapeutic options for inherited cardiovascular diseases in the future [33]. CRISPR/Cas9 can be used to introduce specific variants in hiPSCs, thereby overcoming the need of patient-derived cells and, additionally, enabling the analysis of variants that have not been identified in humans so far. Further, CRISPR/Cas9 provides a highly relevant tool for generating isogenic controls. Although patient-derived hiPSCs are an important model to study the pathophysiology of diseases, in contrast to animal models they lack a constant genetic background and this might influence the observed cellular phenotype. Additionally, the process of reprogramming of somatic cells and generation of iPSC lines itself might induce genetic variations that trigger cellular differences between iPSC lines [34]. To overcome these obstacles and reduce the impact of additional variants on the observed cellular phenotype, it is of particular importance to generate isogenic iPSC lines that differ only in the variants of interest. CRISPR/Cas9-based genome editing enables the creation of such isogenic lines either by introduction of variants of interest into wild-type hiPSCs or by correction of identified disease causing variants in patient-derived hiPSC lines. Both strategies have been employed in the generation and characterization of cellular models of HCM.

Wang et al. used a CRISPR/Cas9-based approach to establish the first hiPSC-CM model of HCM based on mutations in the gene encoding troponin T (*TNNT2*), which is associated with an increased risk of ventricular arrhythmias and sudden death in human [35]. They demonstrated that introduction of the p.Ile79Asn variant in Troponin T recapitulated key cellular findings of HCM, including a high degree of sarcomere disorganization, myofilament disarray, and reduced Ca^2+^ transients, which was caused by an increase in cytosolic Ca^2+^ buffering. Interestingly, they did not observe any difference in cell size and cell volume as seen in other HCM hiPSC-CMs models, and myocardial contractility was increased despite the observed disorganization and shortening of the sarcomere [35]. Applying a CRISPR/Cas9-based editing strategy, Hanses et al. specifically targeted an intronic *LZTR1* variant in hiPSCs derived from a patient with autosomal recessive Noonan syndrome [31]. Deletion of this variant abrogated LZTR1 missplicing and restored the expression of functional LZTR1 from this allele. hiPSC-CMs generated from these corrected hiPSCs exhibited a reduction in cell size and restoration of Ca^2+^ transients, resulting in a cellular phenotype similar to that of wild-type hiPSC-CMs [31]. Recently, a CRISPR/Cas9-based genome editing strategy in hiPSCs was used to determine the effect of a missense variant in MYL3, which was identified in a clinically unremarkable individual and had been annotated as likely pathogenic [36]. Ma et al. used patient-derived hiPSC-CMs carrying this alteration, p.Ala57Asp, in MYL3 in the heterozygous state and CRISPR/Cas9-edited isogenic hiPSC lines harboring either the wild-type or the mutated allele in the homozygous state. They revealed that this missense variant does not cause any cellular changes in, e. g., cell size, sarcomere structure, contractility, action potentials, or calcium handling, therefore making it unlikely to be causative of HCM [36]. Several additional isogenic models for HCM based on CRISPR/Cas9 editing strategies in hiPSCs which further elucidated cellular phenotypes in HCM and revealed differential cellular phenotypes and responses to therapeutic treatments have recently been published [37], [38].

During the last years, hiPSC-CMs have been an important tool for investigating cellular pathomechanisms in HCM. However, hiPSC-CMs display cellular features typical of immature, fetal cardiomyocytes rather than of adult cardiomyocytes. This includes their gene expression profile as well as features of cell morphology, sarcomere arrangement, and energy metabolism [39], [40]. hiPSC-CMs show low expression of several ion channels and proteins involved in Ca^2+^ handling [41], [42]. Their energy generation is mainly dependent on glycolysis instead of fatty acid oxidation seen in adult cardiomyocytes, and they exhibit a higher degree of sarcomere disarray, a tendency to spontaneously contract, and a reduced contractile force [39], [40]. Moreover, most hiPSC-based strategies aim to differentiate hiPSCs into contracting cardiomyocytes. Although cardiomyocytes make up 70–85 % of the volume of cardiac tissue, non-myocytes, like fibroblasts and endothelial cells, are essential for normal heart homeostasis. For example, impairment of cardiac fibroblast function, as seen in CFC syndrome due to pathogenic variants in *BRAF*, contributes to the hypertrophic phenotype of cardiomyocytes through increased TGFβ secretion, indicating that solely focusing on hiPSC-CMs as a disease model does not fully take into account the complexity of the human heart [30]. However, recent advances in the generation of organoids and tissue engineering might solve these problems by providing more appropriate models of the human heart and offering new opportunities to study pathomechanisms and therapeutic options in HCM.

## Engineered models of the human myocardium

Standard monolayer cultures of hiPSC-CMs neither represent the developmental stage and morphologic properties of their mature *in vivo* counterparts, nor are they able to mimic the specific complexity of heart architecture. Hence, additional refined models are urgently needed to better recapitulate the human myocardium in health and disease. The development of microengineered three-dimensional (3D) cardiac tissue models closed this gap not only by providing a more realistic experimental platform for the analysis of human cardiac tissue, but also by possessing the potential to serve as *in vivo* surrogate of the myocardium.

Several strategies have been described to generate 3D cardiac tissue, often relying on hiPSC-CMs, and implementation of additional stimuli has enhanced maturation of hiPSC-CMs in these 3D models. Culturing hiPSC-CMs in different 3D scaffolds for example restored rod-shaped cell morphology of mature cardiomyocytes, induced a fetal-to-adult gene expression shift with increased expression of ion channel-, Ca^2+^ handling-, and sarcoma-related genes, and led to contractile and electrophysiological properties similar to those of mature cardiomyocytes [43], [44], [45], [46], [47], [48]. Maturation of hiPSC-CMs can be further enhanced by electric stimulation, mechanical stimulation like, e. g., mechanical stretch or hydrostatic pressure, or co-culturing of hiPSC-CMs with fibroblasts, all promoting hiPSC-CM maturation and inducing the generation of adult-like, mature human cardiomyocytes [45], [46], [49], [50], [51].

Most bioengineered 3D cardiac tissue models rely on scaffolding structures which promote assembly of hiPSC-CMs and organization of cardiac muscle fibers. *In vivo*, these processes are linked to the extracellular matrix, which plays an essential role in cell differentiation, proliferation, and migration, giving rise to the complex cardiac tissue architecture [52], [53]. *In vitro* 3D cardiac models can substitute this structure either by usage of synthetic polymers as, e. g., polydimethylsiloxane (PDMS), natural polymers like collagen, fibrin, or gelatin, or decellularized heart tissue, e. g., from mice [51], [53], [54]. These engineered 3D cardiac tissue models can further be improved by introducing additional cell types, which leads to a better reflection of the physiological situation in the beating heart *in vivo*. For instance, incorporation of cardiac fibroblasts directly influences maturation of hiPSC-CMs and induces synchronized beating of these cells [55]. Addition of endothelial cells changes hiPSC-CMs’ transcription profiles, inducing the expression of cardiac ion channels and Ca^2+^ handling proteins, both of which are also observed during cardiomyocyte maturation [56].

3D engineered heart models have also become an important tool to investigate pathological heart conditions. Prondzynski et al. showed in a proof-of-principle study that 3D cardiac tissue model derived from hiPSC-CMs of HCM patients can be used to classify variants of unknown significance and to develop personalized treatment options [57]. This is further supported by recent advances in tissue engineering that enable to miniaturize 3D cardiac models to fit into 96-well plates allowing, e. g., automated high-throughput compound screening and drug testing [58].

Finally, these 3D cardiac models not only provide a more physiological *in vitro* model of the heart, but may also serve as surrogate tissue for cardiac repair. As the complexity and functionality of 3D engineered heart tissue are continuously increasing, transplantation of engineered cardiac patches becomes a realistic therapeutic option for cardiac repair, e. g., after acute myocardial infarction [47], [59].

## Conclusion and outlook

In recent years, hiPSC technologies and the enormous progress in deriving cardiomyocytes from these cells have been giving rise to the development of invaluable novel human-based experimental models that help to understand cardiac processes in health and disease. Here, we highlight hiPSC-based cellular models and their role in investigating molecular and cellular processes that contribute to the development of HCM. Ongoing research efforts will further improve current cellular and tissue models by reflecting the *in vivo* situation more accurately and will provide optimized platforms for, e. g., drug testing, tissue regenerative approaches, and personalized medicine. Importantly, 3D cardiac models provide powerful tools for possible clinical applications of genome editing technologies, including CRISPR/Cas9-based approaches, opening up new perspectives for precision medicine and *in vivo* somatic genome editing approaches in HCM, as recently shown for genetic cardiomyopathy-associated disorders [60].
